# Lectures to Ladies

**Published:** 1875-11

**Authors:** 


Lectures to Ladies.—There is a very interesting course of lectures, on Physiology, now being delivered to ladies at Cooper Union, Room 18, by Prof. Adrian J. Ebell, M.D., Director of International Academy. These lectures are illustrated by models, skeletons and the stereopticon, also by drawings upon the black-board, and the Dr. is so thoroughly conversant with his subject, and delineates it in so attractive a manner, that the acquisition of information, so useful and necessary, becomes a delightful entertainment. It is designed to continue these lectures throughout the winter, on Monday and Thursday of each week, at 3½ P.M. The small expense involved brings them within the reach of almost every lady. Six dollars for 16 lectures, and ten dollars for 35 lectures; and for fifty cents a most pleasant hour may be enjoyed by those who have not leisure enough for the whole course.

